# Endoscopic Management of Jejunal Diverticular Bleeding: A Case Report and Systematic Review of the Literature

**DOI:** 10.1155/crgm/1928370

**Published:** 2026-08-02

**Authors:** Marta Pettinelli, Benedetto Neri, Dario Biasutto, Claudia Marinaccio, Giulia Parisi, Leandro Corradino, Margareth Martino, Francesco Maria Di Matteo

**Affiliations:** ^1^ Therapeutic GI Endoscopy Unit, Fondazione Policlinico Universitario Campus Bio-Medico, Rome, Italy; ^2^ Gastroenterology Unit, Università Campus Bio-Medico, Rome, Italy, unicampus.it; ^3^ Medicine and Surgery, Universitá Campus Bio-Medico di Roma, Via Alvaro del Portillo, 21, Roma 00128, Italy; ^4^ Università Campus Bio-Medico, Rome, Italy, unicampus.it

**Keywords:** case report, jejunal diverticulum, small bowel bleeding, urgent endoscopy

## Abstract

Bleeding from a jejunal diverticulum is a rare and potentially severe condition, challenging both to diagnose and to treat. We report the case of a 56‐year‐old woman presenting with massive melena, who initially underwent esophagogastroduodenoscopy (EGD) and abdominal angio‐CT, both unable to identify the bleeding source. After negative urgent ileocolonoscopy, second‐look EGD showed fresh blood in the distal duodenum with no potentially bleeding lesions. This observation prompted us to perform a push enteroscopy that revealed an ulcer with oozing bleeding from a vessel located on the neck of a jejunal diverticulum. Hemostasis was successfully achieved by the placement of 4 through‐the‐scope clips. Currently, device‐assisted enteroscopy (DAE) is the standard technique for the treatment of most causes of small bowel bleeding. However, DAE is performed only by dedicated endoscopists, is not available on a large scale, and is time‐consuming. The present case report suggests the possible role of push enteroscopy as both a diagnostic and therapeutic procedure in the presence of signs suggesting a proximal origin of small bowel bleeding. This technique may indeed avoid unnecessary delay in small bowel bleeding management.

## 1. Introduction

Small bowel bleeding (SBB) is defined as gastrointestinal bleeding originating from a small bowel segment located between the ampulla of Vater and the ileocecal valve. The incidence of SBB ranges from 5% to 10% of all gastrointestinal bleeding [1]. Clinical presentation includes both melena and rectal bleeding, usually associated with hemodynamic instability. The suspicion of SBB is posed after negative upper and lower endoscopy [1].

Small‐bowel capsule endoscopy (SBCE) is the first‐line examination for patients with suspected SBB, due to its ability to visualize the entire small‐bowel mucosa [2]. Enteroscopy refers both to device‐assisted enteroscopy (DAE), which can potentially visualize the entire small bowel, and push‐enteroscopy, which can visualize the small bowel limited to the length of the scope in use. In the bleeding setting, diagnostic enteroscopy is reserved for cases in which SBCE is contraindicated or unavailable, as it is mainly indicated to perform hemostasis once the bleeding source has been identified [3].

The most common cause of SBBs is small bowel angiodysplasia. Other potential causes of SBB include small bowel tumors, ulcers, and Dieulafoy’s lesions [1]. The prevalence of SBB lesions varies according to patient’s age [1]. Bleeding from diverticula other than Meckel diverticulum is rare, accounting for 0.06%–5% of all SBB cases [4].

Small bowel diverticula are pseudodiverticula most frequently located in the duodenum, followed by jejunum and ileum [4, 5]. Jejunal diverticula are rare, with an incidence of 0.4%–4.6%, being most frequent in older patients, with a slight male predominance. Patients with jejunal diverticula are often asymptomatic but susceptible to complications such as hemorrhage, perforation, abscesses, and intestinal obstruction [6]. Bleeding caused by jejunal diverticula can manifest as either acute or chronic and is usually surgically managed, though few cases of successful endoscopic treatment have been reported [4, 5, 7–12].

## 2. Case Presentation

A 56‐year‐old woman presented at the emergency department with melena, severe asthenia, and palpitations. Her medical history included hypertension and bronchial asthma, and she was not on antithrombotic or anticoagulant therapy. Past surgical history included gastric band removal, cholecystectomy, and cesarean section. At presentation, she was hemodynamically stable, but her skin was pale, and the abdomen was diffusely tender. Rectal examination revealed dark blood with no masses. Blood tests showed severe normocytic anemia (hemoglobin 7.2 g/dL), leukocytosis (white cell count 14.400/μL), and raised blood urea nitrogen (83 mg/dL) with normal serum creatinine (0.86 mg/dL).

**FIGURE 1:**
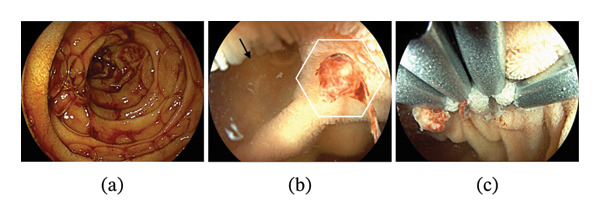
(Panels a–c)**.** Esophagogastroduodenoscopy revealing fresh red blood in the duodenum (Panel (a)). This prompted us to perform push enteroscopy which revealed an ulcer with a visible vessel. (Panel b, centered in a white hexagon) located on the neck of a jejunal diverticulitis (Panel b, black arrow), from which an oozing bleeding originated. Successful hemostasis was achieved by placement of four 13‐mm through‐the‐scope clips (Panel (c)).

Within 12 h from admission, the patient underwent urgent esophagogastroduodenoscopy (EGD), which did not detect any bleeding source. After transfusion with 1 unit of packed red blood cells, due to persistent melena, she underwent an abdominal angio‐CT, which also resulted negative for active bleeding sources. The day after, due to persistent anemia (hemoglobin 6.7 g/dL) and melena despite the infusion of 2 more units of packed red blood cells and 1 g of ferric carboxymaltose, urgent ileocolonoscopy was performed, which did not reveal active bleeding lesions. Dark blood was, however, observed in the terminal ileum, explored for 30 cm. Therefore, during the same procedure, a second‐look EGD was performed, showing fresh red blood in the distal duodenum, in the absence of potentially bleeding sources (Figure 1, panel a). For this reason, we decided to immediately perform an urgent push enteroscopy under propofol sedation, by using a standard intermediate colonoscope (EG‐760R V/I, Fujifilm Corp., Tokyo, Japan). In the proximal jejunum, at approximately 130 cm from the incisors, an 8‐mm ulcer with oozing bleeding from a vessel located on the neck of a diverticulum was detected (Figure 1, Panel b). Therefore, hemostasis was performed by the placement of four 13‐mm through‐the‐scope clips (A.M.I.L. Care, Jiangsu, China) (Figure 1, Panel c). The patient was then hospitalized in an internal medicine ward.

After 48 h of fasting, the patient started oral diet without complications. During hospitalization, a total of 2 more units of packed red blood cells to reach a hemoglobin target > 8 g/dL were administered. Considering the stability of vital signs, the absence of melena, or the lack of further hemoglobin level drop, the patient was discharged 4 days after the endoscopic treatment. At an outpatient follow‐up visit after 6 months, anemia was resolved, and the patient was well.

## 3. Discussion

Small bowel diverticular bleeding can cause severe hemorrhage requiring transfusion, endoscopic intervention, angiography, or surgery [4]. In suspected SBB, SBCE is the first‐line investigation technique, taking into account the excellent safety profile, the tolerability for the patient, and the high diagnostic yield, as it can visualize the entire small‐bowel mucosa. When SBCE is contraindicated or unavailable, diagnostic enteroscopy and/or specific small‐bowel cross‐sectional imaging should be taken into consideration [3]. Indeed, enteroscopy is usually reserved to patients with a diagnosis of SBB amenable to endoscopic treatment. This technique has to be considered for diagnostic purposes only in highly selected cases, including brisk hemorrhage without hemodynamic instability and surgically altered anatomy contraindicating SBCE [3].

**FIGURE 2:**
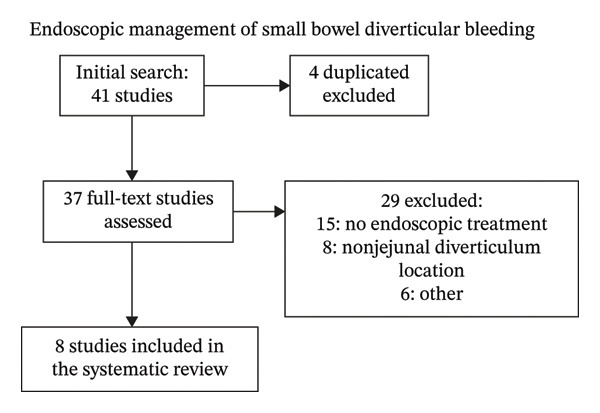
Diagram summarizing the methodology of the literature search leading to the identification of the 8 case reports and case series reporting of endoscopic management of small bowel diverticular bleeding.

In order to provide a comprehensive review regarding the outcomes of patients undergoing endoscopic treatment of SBB originating from a jejunal diverticulum and to compare them to our case, the PubMed and Scopus databases were considered, and the following terms were searched: “jejunal bleeding,” “small bowel bleeding,” “small bowel diverticula,” “jejunal diverticula,” individually or in combination with “Endoscopy,” and “Endoscopic management.” The search was focused on full‐text papers published in English, and no publication date restrictions were used (Figure 2). Forty studies were found, but only studies referring to endoscopically managed SBB from jejunal diverticula were included in the present review, which have been summarized in Table 1.

**TABLE 1:** Summary of currently available case reports of patients with jejunal diverticular bleeding managed by endoscopy.

Year	Authors	Number of patients included	Endoscopic treatment performed	Clinical success (yes/no)	Need for further treatments after endoscopy (type)
2009	Yang et al.	1	Clips	Yes	No
2010	Yen et al.	12	APC, clips	Yes (11/12)	Surgery (1/12)
2010	Chen, et al.	10	Injection therapy, clips	Yes (8/10)	Surgery (2/10)
2011	Chung et al.	1	APC, clips	Yes	No
2015	Chen et al.	35	Injection therapy, clips, APC	Yes (30/35)	Surgery (3/35) Embolization (2/35)
2019	Pinto et al.	1	Clips, adrenaline	No	Surgery
2024	Reed et al.	1	Epinephrine, clips	Yes	No
2024	Angelopous et al.	1	Clips	Yes	No

Abbreviation: APC, argon plasma coagulation.

In the present case, the visualization of fresh blood in the distal duodenum in the absence of potentially bleeding lesions was pivotal to prompt us to proceed with a push enteroscopy. Indeed, as proximal small bowel lesion was strongly suspected, the procedure was performed by using a colonoscope, which is longer and stiffer than a gastroscope, allowing easier access to the jejunum, where the site of bleeding was identified and treated. Indeed, SBCE administration would have delayed both diagnosis and treatment, as it requires the reading time and, for some models, even retrieval of the capsule. Moreover, considering that the patient was actively bleeding, it may have not identified the bleeding source but only the small bowel segment from which it originated. Overall, the risk of retention and the limited visibility of the diverticulum, also due to the volume of blood present, may have impaired the diagnostic yield of SBCE in this particular bleeding setting, as previously reported [5].

Push enteroscopy and DAE are currently not recommended as a first‐line diagnostic procedure in suspected SSB. In particular, discordant recommendations regarding push enteroscopy are reported in the American and European guidelines [1, 3]. Indeed, the American guidelines support the use of push enteroscopy particularly as a second‐look procedure, as it is characterized by a diagnostic yield ranging from 3% to 70% [1]. Differently, European guidelines do not support the adoption of this technique, although acknowledging that the recommendation is based on low‐quality evidence. In the most recent European guideline, it has been suggested that upfront DAE can be indicated in patients at high risk of active SBB, depending on clinical suspicion [3]. However, DAE is performed only by dedicated endoscopists, is not available on a large scale, and is time‐consuming. Thus, in the presence of a strong clinical suspicion or signs of proximal SBB, push enteroscopy could be performed as it allows to perform both diagnostic and therapeutic procedures [3, 13]. Nonetheless, push enteroscopy should not be considered as an alternative to DAE but rather as a procedure to be performed earlier as it is more largely available and less time‐consuming and, when positive, it may avoid further investigations.

Patients with small bowel diverticular bleeding usually present with acute massive melena, although few cases of hematemesis have been reported [5]. Diverticulum formation is considered to be secondary to herniation of mucosa and submucosa through the weakest part of the bowel muscle wall, where blood vessels penetrate, as a result of elevated intraluminal pressure. Possible causes of jejunal diverticular bleeding have been suggested, including ulceration from diverticulitis or trauma to the mesenteric vessels produced by the development of a concretion (enterolith) [5]. Differently, Meckel diverticulum is a true congenital diverticulum, representing one of the most common gastrointestinal malformations. It may contain abnormal tissue (i.e., gastric) and cause bleeding, perforation, or diverticulitis, usually in young patients [14].

Hemostatic enteroscopic procedures rely on the type of lesion identified and include mechanical therapy, cauterization, drug injection, or topical agents [3]. Physiopathology and bleeding characteristics of jejunal diverticula share similarities with colonic diverticula. Therefore, the preferred hemostatic procedures for small bowel diverticular bleeding are shared with that of colonic diverticular bleeding, including through‐ or over‐the‐scope endoclips [15]. In our case, the presence of an actively bleeding diverticular ulcer pushed us to use through‐the‐scope clips as it was the fastest available hemostasis technique, not requiring endoscope retrieval. Successful endoscopic treatment of small bowel diverticular bleeding has been previously reported; however, different hemostatic techniques have been applied. In 2009, Yang et al. reported endoscopic successful treatment of small bowel diverticular bleeding with clip placement [5]. In 2011, Chung et al. performed argon plasma coagulation on reddish spot near jejunal diverticulum. After the procedure, one eroded exposed vessel was observed, and successful hemostasis was performed after placing one clip on the vessel [8]. In 2024, Reed et al. revealed a large jejunal diverticulum with a nonbleeding vessel, which was treated with epinephrine and placement of three clips [7].

If endoscopic hemostasis is not achieved in patients with overt SBB, interventional radiology or surgery should be considered [3]. The preferred treatment for jejunal diverticular bleeding in the past has been resection of the affected bowel [5]. The current technical and technological advancements in enteroscopy may reduce the need for surgery by improving the diagnostic and treatment efficacy, even in rare cases such as jejunal diverticular bleeding.

In conclusion, we reported a rare case of active SBB from a diverticular jejunal ulceration, highlighting the importance of enteroscopy as an alternative to SBCE in selected cases. Moreover, the present case supports that small bowel diverticular bleeding can be treated conservatively by enteroscopy, suggesting the role of this technique as a safe and efficient alternative to surgery.

## Funding

No funding was received for the present study.

Open access publishing facilitated by Universita Campus Bio‐Medico di Roma, as part of the Wiley ‐ CRUI‐CARE agreement.

## Conflicts of Interest

The authors declare no conflicts of interest.

## Data Availability

The data that support the findings of this study are available from the corresponding author upon reasonable request.
